# Streaming current magnetic fields in a charged nanopore

**DOI:** 10.1038/srep36771

**Published:** 2016-11-11

**Authors:** Abraham Mansouri, Peyman Taheri, Larry W. Kostiuk

**Affiliations:** 1 Department of Mechanical Engineering, American University in Dubai, Dubai, 28282, UAE; 2 Matergenics Engineering, Vancouver, BC, V6G 3K3, Canada; 3 Department of Mechanical Engineering, University of Alberta, Edmonton, Alberta, T2G 2G8, Canada

## Abstract

Magnetic fields induced by currents created in pressure driven flows inside a solid-state charged nanopore were modeled by numerically solving a system of steady state continuum partial differential equations, *i.e.*, Poisson, Nernst-Planck, Ampere and Navier-Stokes equations (PNPANS). This analysis was based on non-dimensional transport governing equations that were scaled using Debye length as the characteristic length scale, and applied to a finite length cylindrical nano-channel. The comparison of numerical and analytical studies shows an excellent agreement and verified the magnetic fields density both inside and outside the nanopore. The radially non-uniform currents resulted in highly non-uniform magnetic fields within the nanopore that decay as 1/*r* outside the nanopore. It is worth noting that for either streaming currents or streaming potential cases, the maximum magnetic field occurred inside the pore in the vicinity of nanopore wall, as opposed to a cylindrical conductor that carries a steady electric current where the maximum magnetic fields occur at the perimeter of conductor. Based on these results, it is suggested and envisaged that non-invasive external magnetic fields readouts generated by streaming/ionic currents may be viewed as secondary electronic signatures of biomolecules to complement and enhance current DNA nanopore sequencing techniques.

Voltage driven electrokinetic flows inside solid-state charged nanopores, with promising applications such as DNA sequencing, medical diagnostic, and genetics research, have been an area of intense research for the past three decades^1^,^2^,^3^. The idea of nanopore sensing is to translocate single charged biomolecules, *e.g.*, DNA, RNA, proteins, and peptides, through the nanopore under an applied electric potential to generate unique electronic signals by modulating the ionic currents. The duration and amplitude of such transient ionic currents reveal chemiophysical properties of the biomolecules^4^,^5^. One major drawback of nanopore sequencing is high translocation velocity of biomolecules. Several approaches have been explored to reduce the translocation velocity inside nanopores, for instance, pressure gradients were introduced recently as a new counterbalance driving force. Slowing down translocation velocity by pressure enhances the capabilities of nanopore sensing by enabling the system to distinguish short length DNA molecules without sacrificing the capture rate and signal-to-noise ratios^6^,^7^,^8^,^9^,^10^. Despite these recent advancements in nanopore sequencing, new sensing techniques are necessary to complement transient ionic current readouts for direct label-free sequencing of biomolecules, allowing the ionic current and a secondary signal to be detected simultaneously.

In this work, we contribute to advancing the understanding of the internal and external magnetic fields in charged nanopores created in the cases of pressure driven streaming current and streaming potential. We also suggest and envision that sensing external magnetic fields generated by the ionic currents may be viewed as secondary electronic signatures of biomolecules to non-invasively complement and enhance current DNA nanopore sequencing techniques.

Electrokinetic flows inside nanopores are a coupled problem between the hydrodynamics of electrolyte solutions as described by Navier-Stokes, and the transport of ions by convection, diffusion, and migration as described by Poisson-Nernst-Planck^11^,^12^,^13^,^14^. There is a considerable amount of literature regarding pressure-driven electrokinetic flows in nano and micro-size pores^15^,^16^,^17^,^18^, however the external, as well as the internal non-uniform magnetic fields, induced by such electrokinetic flows in charged nanopores (governed by Ampere’s law) remains unexplored. Streaming currents, similar to electric currents, generate magnetic fields even though these currents flow within a volume of fluid and are not confined to a linear path as in a cylindrical conductor. The system of continuum partial differential equations that captures the detailed physics of magnetic fields generated by moving ion inside a nanopore is consist of Poisson, Nernst-Planck, Ampere and Navier-Stokes equations; together they form the PNPANS system of equations. The main objectives and novelty of the current work are three folds: a) construct a model for the steady state electrokinetic phenomena inside a charged nanopore in streaming current and streaming potential modes using the PNPANS system of equations, b) conduct a series of numerical simulations and analytically verify the external magnetic fields induced by the ions flowing inside a charged nanopore, and c) conduct numerical simulation and analytical verifications of non-uniform internal magnetic fields induced by ions flowing inside a charged nanopore. The paper is organized as follows. In Section 2, a steady state modeling approach is described in terms of a classical geometry, governing equations in non-dimensional forms, pertinent boundary conditions, and the simulation parameters. In Section 3, we discuss the numerical solution methodology and describe the approach that will be used to validate the magnitude of the magnetic field available from steady state analytical results from the idealized geometry of having a channel of infinite length. In Section 4, the model results are presented for streaming current and streaming potential phenomena, and comparisons are made with streaming and conduction current profiles for different pore diameters and pressure differences. Results are then presented for internal and external magnetic fields in a charged nanopore at steady state for a system with a pore diameter such that the electric double layer spans the pore. These solutions are then analyzed in terms of their magnetic fields. Finally, in Section 5, the key conclusions from the present study are summarized.

## Problem Statement

### Geometry

**Figure 1:**
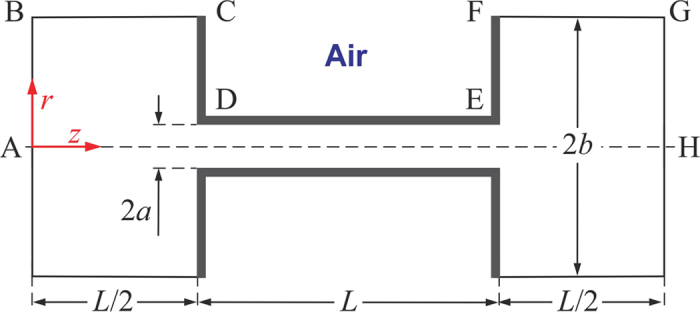
Flow geometry and boundaries of a radially-symmetric modeled finite-length nano-channel connecting two reservoirs. Modeling only one radial section of the nano-channel, A-B and G-H are the inlet and outlet flow boundaries, respectively. D-E is the nano-channel wall, and A-H is the centerline of the flow. C-D and E-F are the front and back faces of the nanopore, respectively. B-C and F-G represent the ends of the computational domain within the inlet and outlet reservoirs, respectively. C-D-E-F defines the thin dielectric material that forms the nanopore. In the current study the thickness of dielectric material is assumed to be zero. The volume contained within C-D-E-F-C is modeled as air. The origin of the coordinate system is placed on the centerline of the inlet boundary. The nano-channel radius is “*a*”, the nano-channel length is “*L*”, and the computed domain of the reservoirs is of radius “*b*” and of length “*L*/2”, respectively.

The flow considered was of an aqueous electrolyte solution passing through a straight circular nano-channel of radius “*a*” and length “*L*” constructed of a dielectric material that connects two reservoirs of radius “*b*” and length “*L*/2”, as shown in Fig. 1. The whole system shown in Fig. 1 will be referred to as the nanopore, while sometimes it will be necessary to restrict discussion specifically to just the channel and that will be referred to as the nano-channel. The surface on the inside of the pore was assumed to have an electrostatic surface potential due to the interaction between the electrolyte and the dielectric. Besides the relative simplicity of the flow, this geometry was used to avoid the formulation of artificial boundary conditions at the nano-channel’s inlet and outlet. This model geometry allows for development of the appropriate composition and flux conditions at the nano-channel entrance and exit that develop out of the “bulk” conditions, which were defined sufficiently far away from nano-channel entrance or exit. Daiguji^17^ first incorporated bulk reservoirs for this purpose in the case of steady state flow, and was subsequently adopted by Mansouri *et al*. for unsteady problems^19^,^20^.

Outside of the liquid flow volume is the confining dielectric material to create walls of thickness “*c*” and “*d*” for the nano-channel and front/back faces, respectively. In this exploratory work, *c* → 0 and *d* → 0 in order to keep the focus on the formation of the magnetic fields and allow for comparison to analytical models. Lastly, the domain defined by C-D-E-F-C in Fig. 1 was assumed to be air, and was considered to capture external magnetic fields that encircle the ion flows inside the nanopore.

### Governing Equations

Electrochemical transport phenomena inside a nanopore were modeled by simultaneously solving the coupled equations of fluid motion (Navier-Stokes) and the ion transport (Poisson-Nernst-Plank)^21^. The hydrodynamic problem was modeled in the framework of the continuity and Navier-Stokes equations. The general form of the steady state momentum equations with an electrical body force, typically used in electrokinetic problems, is


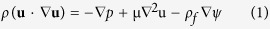


where *ρ* is the fluid density, **u** = (*u, v*) is the velocity vector with *u* and *v* being the radial and axial components, respectively, *p* is pressure, *ρ*_*f*_ is the free electrical charge density (C/m^3^), *ψ* is the electrical potential. In this study there were no external magnetic fields, as well any magnetic fields induced by the small streaming currents were considered to have negligible impact on fluid flows inside nanopore. As a result, the Lorentz force term (*i.e.*, 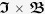, where 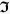 is the ionic current density, and 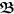 is the magnetic flux density) contribution was not considered^22^.

For calculation of charge distribution, electrical potential, and ion transport, the Poisson-Nernst-Planck (PNP) system of equations was used. The free charge density was related to the electrical potential by the Poisson equation


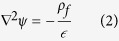


where *ε* is the dielectric permittivity of the fluid, and *ρ*_*f*_ was calculated from 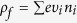 where *e* is the elementary charge, and *υ*_*i*_ and *n*_*i*_ are the valence and number concentration of the *i*^th^ electrolytic species. Ion transport in the electrolyte solution subjected to induced electrical fields was described by the Nernst-Planck equation


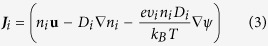


where *D*_*i*_ is the diffusivity of *i*^th^ electrolytic species, *T* is temperature and *k*_*B*_ Boltzmann’s constant. Furthermore, it was assumed that the electrolyte solution is very dilute such that all fluid properties were considered to be uniform and constant throughout the liquid domain. It should be emphasized here that in our simulations, no time-dependent terms were retained in the governing transport equations.

The potential sources of magnetic fields are flows of either electrons or ionic species. In the absence of a time-changing electric flux density, a flowing electric current or in this study an ionic current density 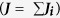 give rise to magnetic fields both inside and around the nanopore. According to Ampere’s circuital law, curl of a magnetic field strength (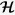) is equal to the current density, such that


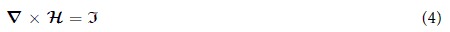


Magnetic field strength is related to magnetic flux density by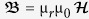, where *μ*_0_ is the vacuum permeability (4π × 10^−7^ N/A^2^). For air the relative permeability *μ*_*r*_ ~ 1. Conservation of current, or the equation of current continuity, is given by ∇·***J*** = 0. The total flux of ions in the solution is represented as a sum of convective, diffusive, and migratory fluxes, as shown in Equation 3.

For pressure driven flows inside charged nanopores there are two limiting steady state scenarios worth considering. The first scenario is the streaming potential mode (*i.e.*, when there is no external electrical connection between the two reservoirs) where pressure driven flow acts tangentially on the mobile portion of electric double layer and generates a streaming current. However, since the external circuit is open, the imbalance of net charges in the vicinity of the entrance and exit of nano-channel induces a streaming potential that generates a conduction current of a proportional magnitude opposite to streaming current. Assuming diffusion current is negligible, the total flux of ions in the solution is given by 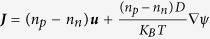, where 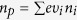 summed only over the positively charged, and *n*_*n*_ is the same but summed over the negative species. The two terms that make up the total flux of ions have opposite directions in the streaming potential mode resulting a zero net current.

The second scenario is the streaming current mode where an external short-circuited electrical connection is made between the reservoirs so that the reservoirs are maintained at the same potential. This is approximated experimentally by placing a standard platinum electrode in each reservoir and connecting them by a wire. Any imbalance of net charge instantaneously induces chemical reactions at electrodes so, no flow induced electrical field will exist in nanopore if polarization of electrodes is neglected. In this mode, the streaming current, which is the product of net charge density and velocity field becomes ***J*** = (*n*_*p*_ − *n*_*n*_)***u*** and is non-zero.

### Non-Dimensional Equations

To facilitate the solution, and to accommodate consideration of the electric double layer effects in the simulations, all the governing equations were non-dimensionalized the same as Mansouri *et al*.^3^, and the characteristic length for this study is chosen to be the Debye length, *κ*^−1^. The definition of the Debye length for a symmetric binary electrolyte is


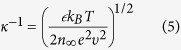


All length parameters are scaled with respect to the Debye length. The scaled parameters used in the present model are shown in Table 1. It should be noted that we also assume the diffusivities of the different ions in the electrolyte solution to be equal (henceforth, *D*_*i*_ = *D*). While it is quite straightforward to incorporate different diffusivities of ions in the numerical model, we use a single diffusivity in our subsequent analysis.

By substituting the non-dimensionalized parameters into the momentum equation, one can obtain the non-dimensional form of the momentum equation


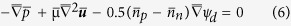


The non-dimensional form of the Poisson equation is


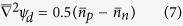


The non-dimensional Nernst-Plank equations for each ionic species for the positive and negative ions, respectively, are:


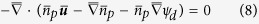



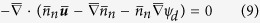


The non-dimensional form of Ampere’s Law is given as


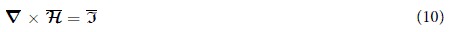


### Boundary Conditions

The boundary conditions applied to the geometry shown in Fig. 1 are based on flow going from left to right. Irrespective of whether the flow considered is to represent streaming current or streaming potential modes the inflow boundary (A-B) was specified at a constant pressure in order to enable a fix total flow rate through the nanopore. The inflow was also an electrically neutral electrolytic solute with a specified ion concentration (*n*_∞_), and at zero electrical potential. The nanopore walls (*i.e.*, C-D, front face; D-E, nano-channel; and E-F, back face) were set impermeable and no-slip for the solvent and solutes. The boundaries shown at *r* = *b (i.e*., B-C and F-G) in the reservoirs are impermeable to solvent and solutes, but have slip conditions to mimic semi-infinity reservoirs. The outflow boundary (G-H) was specified to be at ambient pressure. The nanopore centerline (A-H) was assigned symmetric conditions of zero gradients in all quantities.

For the streaming current mode, instead of modeling the complex electrodes electrochemistry and their polarization, the outflow boundary (G-H) was just specified to be at zero electrical potential and specified ion concentration (neutral electrolyte). For this case the electrical characteristics of nano-channel was given the classical model of a specified surface potential. For the streaming potential mode, zero gradients in electrical potential and concentration of the electrolytes were applied at G-H. For this case the electrical characteristics of nano-cannel was given a specified surface charge density (of a magnitude equivalent to surface potential assigned in the streaming current case if the surface were planar and exposed to the same electrolyte) to allow the local potential to adapt to the potential difference between the two reservoirs.

All of the calculations were performed using a scaled nano-channel length of *κL* = 50, while the scaled radius and length of the reservoirs were both 25. The following physical parameters were employed in the calculations to normalize different quantities according to Table 1: *e* = 1.6021×10^−19^ *C, ρ* = 10^3^ kgm^−3^, *n*_∞_ = 6.022×10^21^ *m*^−3^, 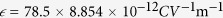, *υ* = 1 *T* = 298 K, *k*_*B*_ = 1.38×10^−23^ JK^−1^, and surface potential for streaming current case = −0.025 V (or surface charge density for streaming potential case = −0.00019 μC/cm^2^), *D* = 10^−9^ *m*^2^*s*^−1^. With the choice of these properties, the Debye length becomes 96 nm. Parameters that were varied in the results section were the channel radius and the upstream reservoir pressure to allow the free charge density in the EDL and flow rate to be altered, which affects the magnitude and spatial distribution of the transport of charge by convection.

## Numerical Methodology and Validation

### Numerical Solution Methodology

In the present work, fully coupled stationary solver integrated in COMSOL (V5.2) finite element software, was used to solve the non-linear partial differential equations. The solution methodology involved a coupled solution of the Poisson-Nernst-Planck (convection, migration, and diffusion), Ampere, and Navier-Stokes (PNPANS) partial differential equations in a 2D axisymmetric computational domain. The finite element calculations were performed using quadratic triangular elements. The accuracy of the numerical results depended strongly on the finite element mesh. Near the nano-channel wall, where the electric field was pronounced, a refined mesh was necessary to ensure accurate modeling of these gradients. Also, near the nanopore centerline, velocities were relatively large and highly dependent on the accuracy of the electrical body force term. Therefore, one needed to have a sufficiently refined mesh to capture the subtle changes in the electric and magnetic fields. Consequently, a finer mesh was used near the nano-channel walls as well as along the center of the nanopore. Independence of the results to mesh refinement was studied and all results reported here are independent of measurable influence of mesh size. It was observed that the solution became mesh independent with approximately 26490 elements.

### Validation

To validate the finite element formulation, we compared the predictions from the steady-state numerical model against existing analytical results for streaming potential flow in an infinitely long nano-channel. The analytical results were obtained for the transport of a symmetric electrolyte in a straight circular cylindrical nanopore of infinite length with low surface potentials on the nanopore wall. Comparisons were made between scaled velocity at the mid-plane of the nanopore (*i.e*., 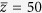), streaming potential across the length of nanopore and analytical solutions based on infinitely long pores. The choice of 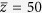 was to be sufficiently far removed from the nanopore entrance and exit regions, and most likely to emulate an infinitely long nano-channel. These velocity comparisons were indistinguishable and in agreement with Mansouri *et al*.^19^. Furthermore both internal and external magnetic fields are in excellent agreements with analytical solutions according to Ampere’s law applied to an infinitely long nano-channel in streaming current case. Details of both these comparisons will be shown in Section 4 – Results and Discussions, while what is described here is the methods used to estimate the magnetic field for the infinitely long nano-channel.

The origin of streaming current is the electrical state of the fluid-substrate interface that creates a spatial distribution in the free charge density, *ρ*_*f*_, which is then transported by the fluid velocity, *v*, parallel to the walls. The streaming current is the product of velocity field and net charge density and is given by


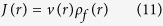


By substituting the velocity field and net charge density from Navier-Stokes and Poisson equations in the absence of any electrical field (*i.e.*, for the streaming current mode) for a circular nanopore with radius “a” we find


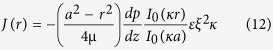


I_0_ is the first kind modified Bessel function, ε is the permittivity, ζ is the surface potential, κ is inverse of Debye length, μ is the viscosity and dp/dz is the pressure gradient. To determine the magnetic fields outside of an infinitely long wire according to Ampere’s law we consider a closed circular loop of radius “r” around the wire, and due to symmetry, magnetic field must have a constant magnitude around the loop


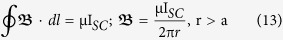


Similarly outside the nanopore, the magnetic field varies as 1/r where *I*_*SC*_ is the streaming current (which is the integral of *J*(*r*) over the whole cross section), r is radial distance from nano-channel wall, *μ* is the permeability and B is magnetic flux density. The magnetic field inside the nanopore however varies nonlinearly with magnetic flux density. This is caused by non-uniform streaming current profiles. Here we show the analytical approach to generate internal magnetic fields. In order to find the magnetic field inside the nanopore, we need to integrate the non-uniform streaming current density for r < R, as shown here


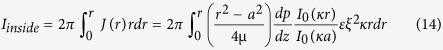


The magnetic field inside the nanopore can be calculated by


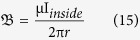


These analytical expressions for the magnetic field arrive from the assumption of infinite pore length, so the results are only a function of radius, and will be compared in the next section to the 2-D numerical results found at the mid-length of the nanopore.

## Results and Discussions

The results presented here are divided into two sections. The first section focuses on the electrokinetics aspects of the flow and set the stage for interpreting the magnet fields that are created due to the currents throughout the nanopore system. The second section focuses on the magnetic fields that exist inside the nanopore system that could affect the transport trajectory of any biomolecules with induced dipoles, and outside the nanopore system that could be monitored as a secondary electromagnetic signal as part of a sequencing technique. As mentioned previously, pressure driven electrokinetic flows in nanopores have two limiting modes of operation, *i.e.* the streaming current mode (no net electrical potential between the reservoirs) and the streaming potential mode (no net current between the reservoirs), and results are presented for both these modes.

### Currents and potentials within electrokinetic nanopores

**Figure 2:**
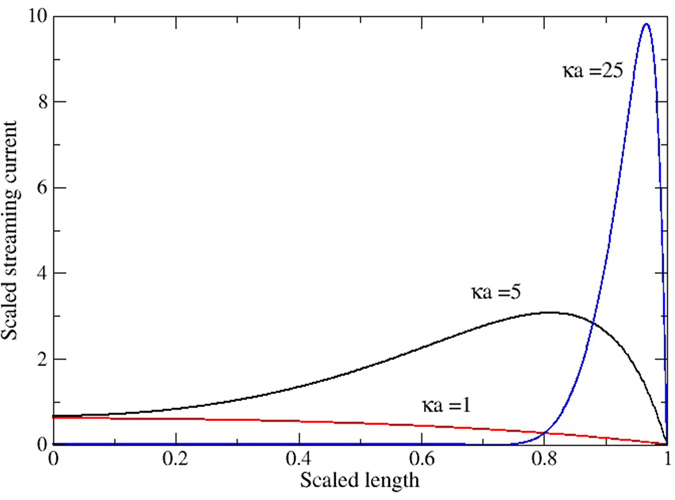
Scaled streaming current profiles in steady state in middle of nanopore (no conduction current). It can be seen that, as κa increases ion transport is dominated by streaming current adjacent to nano-channel wall.

**Figure 3:**
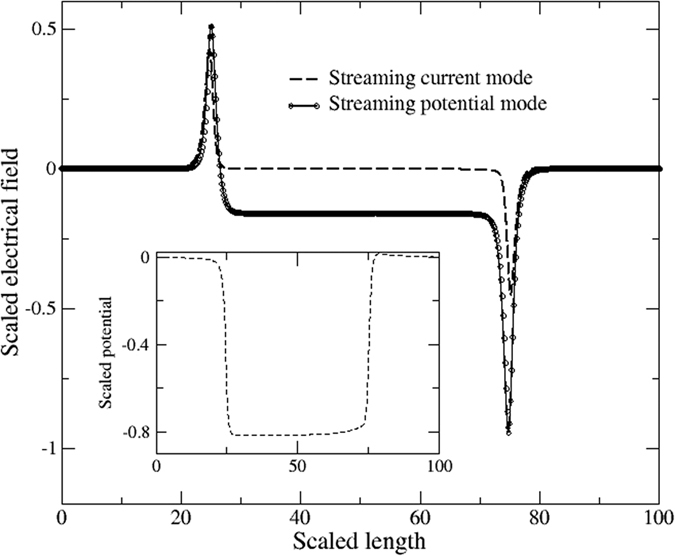
Electrical field inside the nanopore for both streaming current and streaming potential modes. In streaming current mode when both ends of nanopore are short-circuited with ideal electrodes, there is zero electrical field inside the nano-channel that results a zero conduction current. The inset shows the electric potential across the nanopore in streaming current mode.

In the streaming current mode, current in the nano-channel exists due to the convective flux of free charge, and that streaming current is calculated by integrating over the whole cross section of the nano-channel. Figure 2 shows the scaled convection current flux for *κa* = 1, 5 and 25 at the mid-length of the nano-channel for a single pressure gradient of 10^9^ Pa/m. Figure 3 shows the corresponding electrical field along the axis of the nanopores for the particular case of *κa* = 1. The basic observations associated with these two figures are:

1. There is essentially no electric field inside the nano-channel in streaming current mode, but there are spikes in the field of opposite sign that appear at inlet and outlet of the nano-channel. Without the electric field in the nano-channel means that the only mechanism of ion transport is convection.
2. The magnitude and radial distribution of current flux within the nano-channel is dependent on *κa*, as *κa* increases (by varying nano-channel radius) from 1 to 5 and to 25 the non-dimensional current increases from 0.22 pA to 15 pA and to 0.3 nA, respectively. (It should noted that the actual current per unit flow rate drops). For *κa* = 1 the maximum current flux is along the central axis of the nanopore, but as *κa* increases the maximum current flux shifts to nearer the wall.

**Figure 4:**
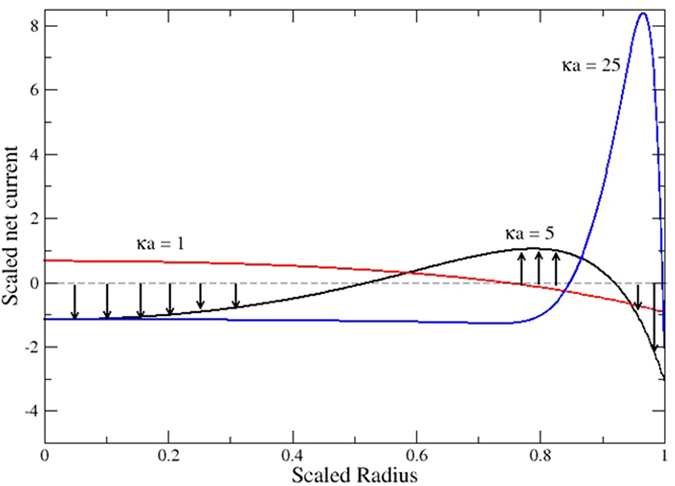
Two-way net current fields in simultaneous conduction and convection currents in steady state in middle of nanopore (zero net current). It can be seen that, for instance, in the case of κa = 5 two regions exist, *i.e.* adjacent to wall and in center where ion transport is dominated by conduction current while between those regions is where the convection current in the opposite dominates.

Also shown in Fig. 3 is the electric field for the streaming potential mode. While this mode has almost identical spikes at the inlet and outlet of the nano-channel, there is a finite and uniform field along the length of the nano-channel. The magnitude of electrical field (0.42 × 10^5^ V/m) is strongly dependent on the pressure gradient and *κa*, and is shown here only for the same pressure gradient (10^9^ Pa/m) and *κa* = 1. This electric field induces a conduction current of the ions, which in steady state balances the convection current. Figure 4 shows the scaled net current flux in the streaming potential mode for *κa* = 1, 5 and 25 at the mid-length of the nano-channel. The key observation associated with Fig. 4 is that while the net current (*i.e.*, the current flux integrated over any cross-section in the nano-channel) is zero, the flux is highly non-uniform. For *κa* = 1, the inner three quarters of the radius has a positive current flux and the outer quarter is negative. While for the *κa* = 25 case, the inner 85% of the radius has a negative current flux there is a significant positive flux near the nano-channel wall^23^.

All of these different spatially different current fluxes, as well as the magnitude of the net current, have important implications for the magnetic fields that exist either within and external to the nanopore.

### Magnetic fields within and around electrokinetic nanopores

**Figure 5:**
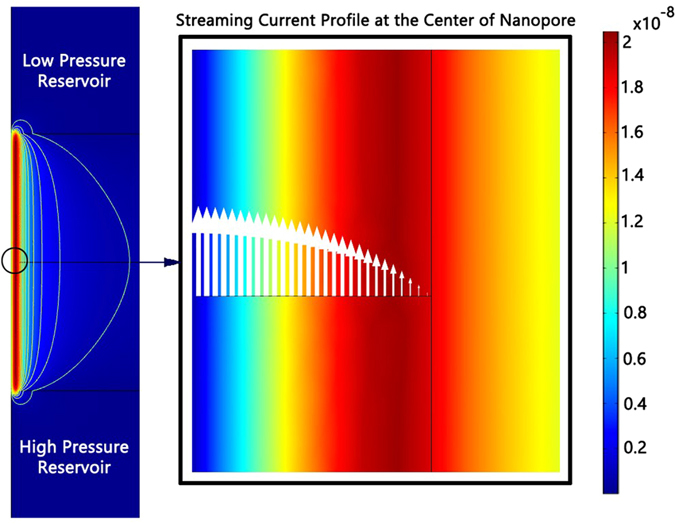
Left: Magnetic field strength (H) profiles and contours induced by streaming currents inside and around the nanopore. As expected the magnetic field decays rapidly outside the nanopore. Right: shows a magnified view of nanopore, white arrows show the profile of streaming current in middle of nanopore at κa=1.

We now turn our attention to magnetic fields induced by the various current fluxes presented in the previous section. Figure 5 shows a 2-D plot of the magnetic field strength for the case of streaming current and κa = 1 for a pressure gradient of 10^8^ Pa/m. The important qualitative observations are that, other than very near the inlet and outlet of the nano-channel, the gradients in the magnetic field strength are only a function of the radial coordinate, that maximum value of the magnetic field exist near the wall of the nano-channel, and extends outside the nano-channel where it can potentially be measured. Overlaid onto the magnetic field strength are vectors with magnitudes scaled to the local current flux, which shows that while the total current inside any radius continues to increase until the full radius is reach, the magnetic field strength reaches a maximum within the nano-channel because of the 1/r dependency from the various convection current streaming tubes.

**Figure 6:**
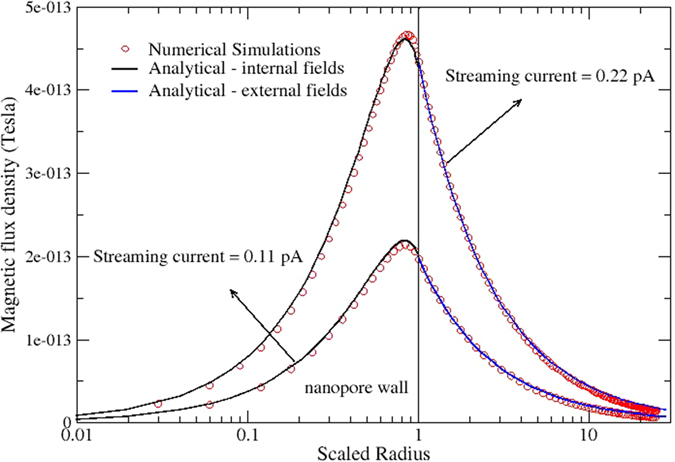
Magnetic flux density inside and outside nanopore’s wall for two different sets of boundary conditions for streaming current case. Numerical simulations include the solution of PNPANS equations i.e. Poisson-Nernst-Planck, and Ampere and Stokes. Analytical solutions for inside the nanopore fields obtained by the integration of non-uniform streaming current profiles.

**Figure 7:**
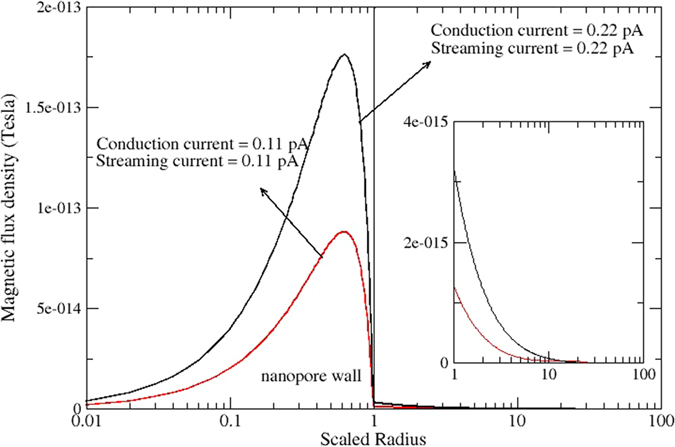
Numerical simulation of magnetic flux density inside and outside nanopore’s wall for two different sets of boundary conditions for steady state streaming potential case (zero net current). The inset shows magnified view of external magnetic fields.

More quantitative measures of the magnetic field are shown in Figs 6 and 7 for the streaming current and streaming potential modes, respectively. These figures are the radial profile of the magnetic flux density on a log scale for *κa* = 1 at the mid-length of the nano-channel, and for two different pressure gradients (5 × 10^8^ and 10^9^ Pa/m, which produce convection currents of 0.11 and 0.22 pA, respectively). The log scale is necessary to capture the range of spatial gradients. Before discussing these results it is worth comparing the magnitude of these fields with the analytical results of the idealized case of an infinitely long channel described in section 3.2 for streaming current case. In Fig. 6 there is excellent agreement between the numerical and analytical results at this location in the nano-channel. In reference to Fig. 5, it is clear that this level of agreement is due to making the comparison at the mid-length of a relatively long the nano-channel.

With respect to the magnetic flux density for *κa* = 1, it is noted that its peak value in all case is very close to the nano-channel wall that decays to zero at the centerline (symmetry requirement) and in the far field (as 1/*r*). Since there is no net current in the streaming current mode, the magnitude of the magnetic field decays at a relatively modest rate compared to the streaming potential case with zero net current across the nano-channel. It’s interesting to note that due to non-uniform distribution of net current in the center of nano-channel in streaming potential case (Fig. 4), internal and external magnetic fields are not zero, however external magnetic fields are notably lower than the streaming current case in Fig. 6.

With the existence of these magnetic fields two possible interactions with molecules that can have an induced dipole are envisioned. First, Zhai *et al*. has shown the external magnetic fields as small as pico Tesla, which is of the order that was modeled here, are detectable and measurable in voltage/pressure driven electrokinetic flow in nanopores^24^,^25^,^26^. As a result, we envision that non-invasive external magnetic fields readouts generated by streaming/ionic currents may be viewed as secondary electronic signatures of biomolecules to complement and enhance current DNA nanopore sequencing techniques. Second, depending on the trajectory that a biomolecule enters the nano-channel it could be displaced to a preferred radial location and become subjected to that local axial flow velocity. As a result, the magnetic fields generated by these electrokinetic flows could be used as a separate management tool for the transport of biomolecules. Our postulate is also supported by experimental evidences in the field of biology and medicine. It is known that several tissues and living organ systems in human body that are electrically excitable induce external magnetic fields. Therefore, bio-magnetic field measurements employing magnetic sensors with high sensitivity provide a non-invasive detection of living system activity. Most recent advances in magnetic sensors technology include a superconducting quantum interference device (SQUID) with the sensitivity of a femto tesla (fT) to detect magnetic activity in the brain and heart of humans^27^.

## Conclusions

In this study, a set of PNPANS equations, consisting of Poisson, Nernst-Planck, Ampere and Navier-Stokes equations were solved numerically to simulate induced internal and external magnetic fields created by streaming current/potentials inside a charged nanopore at κa = 1. The magnetic fields outside the nanopore decays rapidly and varies with inverse of distance according the Ampere’s law. However the magnetic fields inside the nanopore are highly non-uniform. The maximum of magnetic field occurs inside the nanopore and in the vicinity of nanopore wall contrary to magnetic fields of electric currents inside conductive wires. We suggest that non-invasive external magnetic fields readouts generated by streaming/ionic currents are of great importance and may be viewed as secondary electronic signatures of biomolecules to complement and enhance current nanopore sequencing techniques.

## Additional Information

**How to cite this article**: Mansouri, A. *et al*. Streaming current magnetic fields in a charged nanopore. *Sci. Rep.* **6**, 36771; doi: 10.1038/srep36771 (2016).

**Publisher’s note:** Springer Nature remains neutral with regard to jurisdictional claims in published maps and institutional affiliations.

## Figures and Tables

**Table 1:** Non-Dimensional and Scaled Parameters.

Radial Coordinate	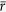	*κr*
Stream-Wise Coordinate	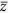	*κ*z
Velocity	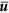	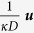
Density	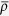	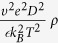
Pressure	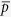	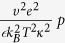
Viscosity	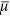	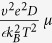
Electrical Potential	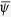	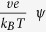
Free Charge Density	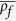	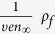
Ion Concentration	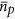 , 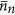	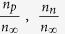
Current	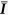	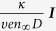
Current density	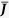	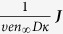
Magnetic Field Density	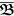	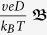
Magnetic Field Strength	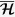	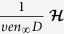
